# Bis[2-(1*H*-benzimidazol-2-yl)acetato-κ^2^ *N* ^3^,*O*]bis­(ethanol-κ*O*)nickel(II)

**DOI:** 10.1107/S160053681204514X

**Published:** 2012-11-07

**Authors:** Jun Wang, Jian-Hua Nie

**Affiliations:** a Zhongshan Polytechnic, Zhongshan, Guangdong 528404, People’s Republic of China

## Abstract

In the title compound, [Ni(C_9_H_7_N_2_O_2_)_2_(C_2_H_5_OH)_2_], the Ni^II^ ion is situated on an inversion center and is coordinated by two N and two O atoms from two 2-(1*H*-benzimidazol-2-yl)acetate (*L*) ligands and by two O atoms from two ethanol ligands in a distorted octa­hedral geometry. In the *L* ligand, the acetate group deviates significantly from the benzimidazole plane, the C—C—C—O(coordinating) torsion angle being 34.2 (5)°. In the crystal, O—H⋯O and N—H⋯O hydrogen bonds link the mol­ecules into a two-dimensional supra­molecular network parallel to the *bc* plane.

## Related literature

For related structures, see: Chen *et al.* (2010 ▶); Gao *et al.* (2011 ▶); Guo *et al.* (2007 ▶); Peng *et al.* (2010 ▶).
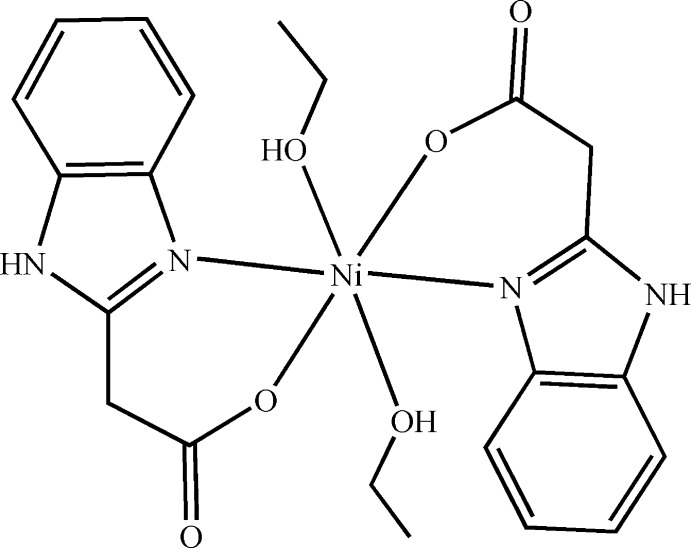


## Experimental

### 

#### Crystal data

[Ni(C_9_H_7_N_2_O_2_)_2_(C_2_H_6_O)_2_]
*M* *_r_* = 501.18
Monoclinic, 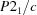

*a* = 10.441 (5) Å
*b* = 9.639 (4) Å
*c* = 11.480 (5) Å
β = 98.956 (6)°
*V* = 1141.3 (9) Å^3^
*Z* = 2
Mo *K*α radiation
μ = 0.90 mm^−1^
*T* = 298 K
0.28 × 0.26 × 0.23 mm

#### Data collection

Bruker APEXII CCD area-detector diffractometer
Absorption correction: multi-scan (*SADABS*; Bruker, 2004 ▶) *T*_min_ = 0.788, *T*_max_ = 0.821
6022 measured reflections
2231 independent reflections
1411 reflections with *I* > 2σ(*I*)
*R*_int_ = 0.061

#### Refinement

*R*[*F* ^2^ > 2σ(*F* ^2^)] = 0.049
*wR*(*F* ^2^) = 0.107
*S* = 1.04
2231 reflections
152 parameters
H-atom parameters constrained
Δρ_max_ = 0.36 e Å^−3^
Δρ_min_ = −0.45 e Å^−3^

### 

Data collection: *APEX2* (Bruker, 2004 ▶); cell refinement: *SAINT* (Bruker, 2004 ▶); data reduction: *SAINT*; program(s) used to solve structure: *SHELXS97* (Sheldrick, 2008 ▶); program(s) used to refine structure: *SHELXL97* (Sheldrick, 2008 ▶); molecular graphics: *ORTEPIII* (Burnett & Johnson, 1996 ▶) and *PLATON* (Spek, 2009 ▶); software used to prepare material for publication: *SHELXL97*.

## Supplementary Material

Crystal structure: contains datablock(s) I, global. DOI: 10.1107/S160053681204514X/cv5355sup1.cif

Structure factors: contains datablock(s) I. DOI: 10.1107/S160053681204514X/cv5355Isup2.hkl

Additional supplementary materials:  crystallographic information; 3D view; checkCIF report

## Figures and Tables

**Table 1:** Hydrogen-bond geometry (Å, °)

*D*—H⋯*A*	*D*—H	H⋯*A*	*D*⋯*A*	*D*—H⋯*A*
O3—H3*A*⋯O2^i^	0.85	1.96	2.672 (3)	141
N2—H2*A*⋯O2^ii^	0.86	1.93	2.788 (4)	173

Symmetry codes: (i) 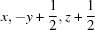; (ii) 
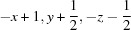
.

**Fig. 1.**
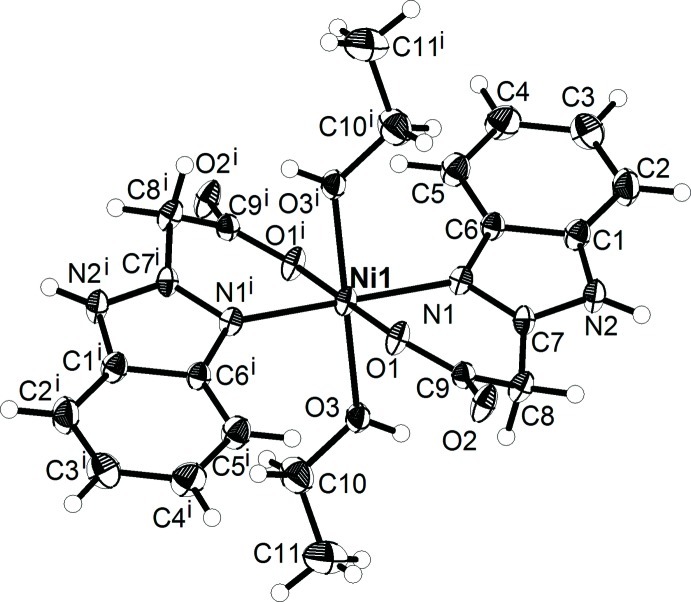
The molecular structure of (I) with displacement ellipsoids drawn at the 30% probability level [symmetry code\: (i) 1 - x, -y, -z].

**Fig. 2.**
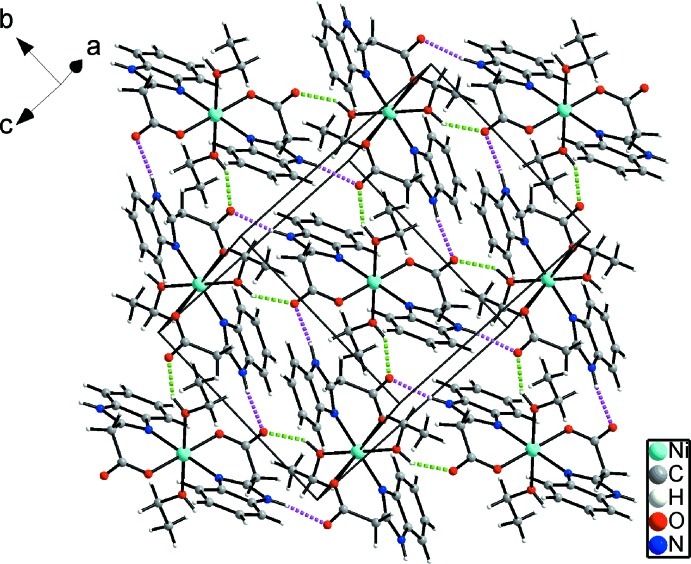
A portion of the crystal packing, showing the two-dimensional supramolecular network. Hydrogen bonds are shown as dashed lines.

## supplementary crystallographic information

### Comment

Multidentate ligands containing N donors and carboxylic groups are often employed to construct new metal coordination polymers with different structures (Chen *et al.*, 2010; Gao *et al.*, 2011; Guo *et al.*, 2007; Peng *et al.*, 2010). The main reason is that they have various coordination modes and can form high-dimensional polymers through hydrogen-bonding interactions in the process of self-assembly. In this work, we chose 2-(1*H*-benzimidazol-2-yl)acetic acid (H*L*), which contains two N atoms of an imidazole group and one carboxylate group, as the building block to prepare new metal coordination polymers. To date, only three mononuclear complexes based on the H*L* ligand have been reported (Chen *et al.*,2010). In this paper, we report the synthesis and structure of the title compound, (I), obtained by the solvothermal reaction of NiCl_2_ and H*L* ligand.

In (I) (Fig. 1), the Ni(II) ion is coordinated by two N and two O atoms from two bidentate chelating *L* ligands and two O atoms from two ethanol molecules in a distorted octahedral geometry. The Ni—N bond length is equal to 2.055 (3) Å, and the Ni—O distances vary from 2.037 (3) to 2.107 (2) Å. In the crystal, intermolecular O—H···O and N–H···O hydrogen bonds (Table1) involving the carboxylate O atoms, the imidazole N atoms and the coordinated ethanol O atoms link the molecules into a two-dimensional supramolecular network parallel to the *bc* plane (Fig. 2).

### Experimental

A mixture of NiCl_2_ (0.40 mmol), H*L* (0.40 mmol) and 8 ml C_2_H_5_OH was sealed into a 15 ml Teflon-lined stainless steel autoclave and then heated at 373 K for 72 h under autogenous pressure. After cooling to room temperature at a rate of 2 K /h, green block crystals of the title compound suitable for X-ray diffraction were obtained (yield: 35%).

### Refinement

C– and N-bound H atoms were positioned geometrically and refined using a riding model, with C—H = 0.93–0.97 Å, N—H = 0.86 Å and with *U*_iso_(H) = 1.2(1.5 for methyl)*U*_eq_(C), *U*_iso_(H) = 1.2*U*_eq_(N). Hydroxy H atoms were located in a difference Fourier map and refined as riding, with O—H = 0.85 Å and *U*_iso_(H)= 1.2*U*_eq_(O).

### Crystal data

**Table:**

[Ni(C_9_H_7_N_2_O_2_)_2_(C_2_H_6_O)_2_]	*F*(000) = 524
*M**_r_* = 501.18	*D*_x_ = 1.458 Mg m^−^^3^
Monoclinic, *P*2_1_/*c*	Mo *K*α radiation, λ = 0.71073 Å
Hall symbol: -P 2ybc	Cell parameters from 1078 reflections
*a* = 10.441 (5) Å	θ = 2.8–21.1°
*b* = 9.639 (4) Å	µ = 0.90 mm^−^^1^
*c* = 11.480 (5) Å	*T* = 298 K
β = 98.956 (6)°	Block, green
*V* = 1141.3 (9) Å^3^	0.28 × 0.26 × 0.23 mm
*Z* = 2	

### Data collection

**Table:**

Bruker APEXII CCD area-detector diffractometer	2231 independent reflections
Radiation source: fine-focus sealed tube	1411 reflections with *I* > 2σ(*I*)
Graphite monochromator	*R*_int_ = 0.061
phi and ω scans	θ_max_ = 26.0°, θ_min_ = 2.8°
Absorption correction: multi-scan (*SADABS*; Bruker, 2004)	*h* = −8→12
*T*_min_ = 0.788, *T*_max_ = 0.821	*k* = −11→11
6022 measured reflections	*l* = −14→14

### Refinement

**Table:**

Refinement on *F*^2^	Primary atom site location: structure-invariant direct methods
Least-squares matrix: full	Secondary atom site location: difference Fourier map
*R*[*F*^2^ > 2σ(*F*^2^)] = 0.049	Hydrogen site location: inferred from neighbouring sites
*wR*(*F*^2^) = 0.107	H-atom parameters constrained
*S* = 1.04	*w* = 1/[σ^2^(*F*_o_^2^) + (0.0364*P*)^2^] where *P* = (*F*_o_^2^ + 2*F*_c_^2^)/3
2231 reflections	(Δ/σ)_max_ < 0.001
152 parameters	Δρ_max_ = 0.36 e Å^−^^3^
0 restraints	Δρ_min_ = −0.45 e Å^−^^3^

### Special details

**Table:**

Geometry. All e.s.d.'s (except the e.s.d. in the dihedral angle between two l.s. planes) are estimated using the full covariance matrix. The cell e.s.d.'s are taken into account individually in the estimation of e.s.d.'s in distances, angles and torsion angles; correlations between e.s.d.'s in cell parameters are only used when they are defined by crystal symmetry. An approximate (isotropic) treatment of cell e.s.d.'s is used for estimating e.s.d.'s involving l.s. planes.
Refinement. Refinement of *F*^2^ against ALL reflections. The weighted *R*-factor *wR* and goodness of fit *S* are based on *F*^2^, conventional *R*-factors *R* are based on *F*, with *F* set to zero for negative *F*^2^. The threshold expression of *F*^2^ > σ(*F*^2^) is used only for calculating *R*-factors(gt) *etc*. and is not relevant to the choice of reflections for refinement. *R*-factors based on *F*^2^ are statistically about twice as large as those based on *F*, and *R*- factors based on ALL data will be even larger.

### Fractional atomic coordinates and isotropic or equivalent isotropic displacement parameters (Å^2^)

**Table:**

	*x*	*y*	*z*	*U*_iso_*/*U*_eq_	
Ni1	0.5000	0.0000	0.0000	0.0295 (2)	
C7	0.5632 (4)	0.2871 (4)	−0.0656 (3)	0.0303 (9)	
C1	0.7327 (4)	0.3710 (4)	0.0516 (3)	0.0372 (10)	
C6	0.7025 (4)	0.2385 (4)	0.0887 (3)	0.0310 (9)	
C8	0.4525 (4)	0.2834 (4)	−0.1641 (3)	0.0357 (10)	
H8A	0.4655	0.3559	−0.2197	0.043*	
H8B	0.3741	0.3059	−0.1325	0.043*	
C5	0.7763 (4)	0.1803 (4)	0.1874 (4)	0.0451 (11)	
H5	0.7568	0.0929	0.2141	0.054*	
C4	0.8787 (4)	0.2548 (5)	0.2444 (4)	0.0564 (13)	
H4	0.9294	0.2178	0.3109	0.068*	
C2	0.8367 (4)	0.4466 (4)	0.1082 (4)	0.0538 (12)	
H2	0.8568	0.5341	0.0821	0.065*	
C3	0.9081 (4)	0.3863 (4)	0.2039 (4)	0.0574 (13)	
H3	0.9789	0.4340	0.2440	0.069*	
C9	0.4305 (4)	0.1481 (4)	−0.2311 (3)	0.0302 (9)	
O2	0.3884 (3)	0.1537 (2)	−0.3388 (2)	0.0435 (7)	
O1	0.4545 (2)	0.0357 (2)	−0.1765 (2)	0.0357 (7)	
N1	0.5959 (3)	0.1869 (3)	0.0125 (3)	0.0300 (8)	
N2	0.6410 (3)	0.3986 (3)	−0.0456 (3)	0.0382 (8)	
H2A	0.6347	0.4739	−0.0864	0.046*	
O3	0.3382 (2)	0.1147 (2)	0.0343 (2)	0.0367 (7)	
H3A	0.3398	0.2025	0.0416	0.044*	
C10	0.2225 (5)	0.0637 (5)	0.0670 (5)	0.0627 (14)	
H10A	0.2386	0.0401	0.1502	0.075*	
H10B	0.1984	−0.0209	0.0233	0.075*	
C11	0.1146 (5)	0.1594 (6)	0.0467 (6)	0.112 (2)	
H11A	0.1364	0.2427	0.0913	0.168*	
H11B	0.0397	0.1175	0.0710	0.168*	
H11C	0.0962	0.1817	−0.0358	0.168*	

### Atomic displacement parameters (Å^2^)

**Table:**

	*U*^11^	*U*^22^	*U*^33^	*U*^12^	*U*^13^	*U*^23^
Ni1	0.0404 (5)	0.0212 (4)	0.0268 (4)	−0.0017 (3)	0.0044 (3)	0.0004 (3)
C7	0.039 (3)	0.022 (2)	0.031 (2)	−0.0052 (18)	0.0072 (18)	−0.0016 (17)
C1	0.045 (3)	0.029 (2)	0.037 (2)	−0.002 (2)	0.003 (2)	−0.0036 (18)
C6	0.035 (3)	0.027 (2)	0.031 (2)	−0.0018 (18)	0.0041 (19)	−0.0012 (17)
C8	0.050 (3)	0.024 (2)	0.031 (2)	0.0009 (19)	0.000 (2)	0.0009 (17)
C5	0.052 (3)	0.037 (2)	0.044 (3)	−0.003 (2)	−0.002 (2)	−0.002 (2)
C4	0.057 (4)	0.054 (3)	0.051 (3)	−0.001 (3)	−0.013 (2)	0.000 (2)
C2	0.059 (3)	0.038 (3)	0.060 (3)	−0.015 (2)	−0.002 (3)	0.004 (2)
C3	0.053 (3)	0.049 (3)	0.065 (3)	−0.014 (3)	−0.007 (3)	−0.015 (3)
C9	0.032 (2)	0.027 (2)	0.032 (2)	−0.0036 (18)	0.0043 (18)	−0.0002 (18)
O2	0.071 (2)	0.0274 (15)	0.0280 (16)	−0.0006 (14)	−0.0061 (14)	0.0026 (12)
O1	0.0565 (19)	0.0214 (14)	0.0283 (15)	0.0004 (12)	0.0041 (12)	0.0010 (11)
N1	0.038 (2)	0.0250 (17)	0.0270 (18)	−0.0005 (15)	0.0042 (15)	0.0012 (14)
N2	0.051 (2)	0.0258 (18)	0.037 (2)	−0.0079 (16)	0.0018 (17)	0.0063 (15)
O3	0.0414 (18)	0.0256 (14)	0.0448 (17)	−0.0020 (13)	0.0118 (13)	−0.0006 (12)
C10	0.058 (4)	0.051 (3)	0.083 (4)	−0.007 (3)	0.025 (3)	−0.014 (3)
C11	0.054 (4)	0.089 (5)	0.195 (8)	0.013 (4)	0.025 (4)	0.012 (5)

### Geometric parameters (Å, º)

**Table:**

Ni1—O1^i^	2.037 (3)	C5—H5	0.9300
Ni1—O1	2.037 (3)	C4—C3	1.400 (6)
Ni1—N1^i^	2.055 (3)	C4—H4	0.9300
Ni1—N1	2.055 (3)	C2—C3	1.358 (6)
Ni1—O3^i^	2.107 (2)	C2—H2	0.9300
Ni1—O3	2.107 (2)	C3—H3	0.9300
C7—N1	1.325 (4)	C9—O2	1.247 (4)
C7—N2	1.346 (4)	C9—O1	1.257 (4)
C7—C8	1.487 (5)	N2—H2A	0.8600
C1—N2	1.379 (5)	O3—C10	1.409 (5)
C1—C2	1.384 (5)	O3—H3A	0.8500
C1—C6	1.398 (5)	C10—C11	1.447 (6)
C6—C5	1.386 (5)	C10—H10A	0.9700
C6—N1	1.395 (5)	C10—H10B	0.9700
C8—C9	1.514 (5)	C11—H11A	0.9600
C8—H8A	0.9700	C11—H11B	0.9600
C8—H8B	0.9700	C11—H11C	0.9600
C5—C4	1.367 (5)		
			
O1^i^—Ni1—O1	180.0	C5—C4—H4	119.6
O1^i^—Ni1—N1^i^	87.54 (10)	C3—C4—H4	119.6
O1—Ni1—N1^i^	92.46 (10)	C3—C2—C1	116.5 (4)
O1^i^—Ni1—N1	92.46 (10)	C3—C2—H2	121.7
O1—Ni1—N1	87.54 (10)	C1—C2—H2	121.7
N1^i^—Ni1—N1	180.0	C2—C3—C4	122.3 (4)
O1^i^—Ni1—O3^i^	91.58 (10)	C2—C3—H3	118.8
O1—Ni1—O3^i^	88.42 (10)	C4—C3—H3	118.8
N1^i^—Ni1—O3^i^	85.58 (11)	O2—C9—O1	123.0 (3)
N1—Ni1—O3^i^	94.42 (11)	O2—C9—C8	117.9 (3)
O1^i^—Ni1—O3	88.42 (10)	O1—C9—C8	119.1 (3)
O1—Ni1—O3	91.58 (10)	C9—O1—Ni1	129.7 (2)
N1^i^—Ni1—O3	94.42 (11)	C7—N1—C6	105.1 (3)
N1—Ni1—O3	85.58 (11)	C7—N1—Ni1	121.3 (2)
O3^i^—Ni1—O3	180.0	C6—N1—Ni1	133.5 (2)
N1—C7—N2	112.5 (3)	C7—N2—C1	107.9 (3)
N1—C7—C8	125.8 (3)	C7—N2—H2A	126.0
N2—C7—C8	121.7 (3)	C1—N2—H2A	126.0
N2—C1—C2	132.4 (4)	C10—O3—Ni1	127.8 (2)
N2—C1—C6	105.2 (3)	C10—O3—H3A	109.0
C2—C1—C6	122.4 (4)	Ni1—O3—H3A	122.5
C5—C6—N1	131.0 (3)	O3—C10—C11	114.4 (4)
C5—C6—C1	119.7 (4)	O3—C10—H10A	108.7
N1—C6—C1	109.3 (3)	C11—C10—H10A	108.7
C7—C8—C9	116.4 (3)	O3—C10—H10B	108.7
C7—C8—H8A	108.2	C11—C10—H10B	108.7
C9—C8—H8A	108.2	H10A—C10—H10B	107.6
C7—C8—H8B	108.2	C10—C11—H11A	109.5
C9—C8—H8B	108.2	C10—C11—H11B	109.5
H8A—C8—H8B	107.3	H11A—C11—H11B	109.5
C4—C5—C6	118.2 (4)	C10—C11—H11C	109.5
C4—C5—H5	120.9	H11A—C11—H11C	109.5
C6—C5—H5	120.9	H11B—C11—H11C	109.5
C5—C4—C3	120.8 (4)		
			
N2—C1—C6—C5	−178.9 (3)	N2—C7—N1—Ni1	−177.1 (2)
C2—C1—C6—C5	1.7 (6)	C8—C7—N1—Ni1	4.1 (5)
N2—C1—C6—N1	1.4 (4)	C5—C6—N1—C7	179.3 (4)
C2—C1—C6—N1	−178.0 (4)	C1—C6—N1—C7	−1.1 (4)
N1—C7—C8—C9	−41.6 (5)	C5—C6—N1—Ni1	−3.7 (6)
N2—C7—C8—C9	139.7 (3)	C1—C6—N1—Ni1	175.9 (2)
N1—C6—C5—C4	178.5 (4)	O1^i^—Ni1—N1—C7	−154.3 (3)
C1—C6—C5—C4	−1.1 (6)	O1—Ni1—N1—C7	25.7 (3)
C6—C5—C4—C3	−0.1 (7)	O3^i^—Ni1—N1—C7	114.0 (3)
N2—C1—C2—C3	179.8 (4)	O3—Ni1—N1—C7	−66.0 (3)
C6—C1—C2—C3	−1.0 (7)	O1^i^—Ni1—N1—C6	29.2 (3)
C1—C2—C3—C4	−0.2 (7)	O1—Ni1—N1—C6	−150.8 (3)
C5—C4—C3—C2	0.8 (7)	O3^i^—Ni1—N1—C6	−62.6 (3)
C7—C8—C9—O2	−146.1 (4)	O3—Ni1—N1—C6	117.4 (3)
C7—C8—C9—O1	34.2 (5)	N1—C7—N2—C1	0.5 (4)
O2—C9—O1—Ni1	−171.0 (3)	C8—C7—N2—C1	179.4 (3)
C8—C9—O1—Ni1	8.7 (5)	C2—C1—N2—C7	178.1 (4)
N1^i^—Ni1—O1—C9	145.8 (3)	C6—C1—N2—C7	−1.2 (4)
N1—Ni1—O1—C9	−34.2 (3)	O1^i^—Ni1—O3—C10	−73.1 (3)
O3^i^—Ni1—O1—C9	−128.7 (3)	O1—Ni1—O3—C10	106.9 (3)
O3—Ni1—O1—C9	51.3 (3)	N1^i^—Ni1—O3—C10	14.3 (3)
N2—C7—N1—C6	0.4 (4)	N1—Ni1—O3—C10	−165.7 (3)
C8—C7—N1—C6	−178.4 (4)	Ni1—O3—C10—C11	−160.5 (4)

Symmetry code: (i) −*x*+1, −*y*, −*z*.

### Hydrogen-bond geometry (Å, º)

**Table:**

*D*—H···*A*	*D*—H	H···*A*	*D*···*A*	*D*—H···*A*
O3—H3*A*···O2^ii^	0.85	1.96	2.672 (3)	141
N2—H2*A*···O2^iii^	0.86	1.93	2.788 (4)	173

Symmetry codes: (ii) *x*, −*y*+1/2, *z*+1/2; (iii) −*x*+1, *y*+1/2, −*z*−1/2.
